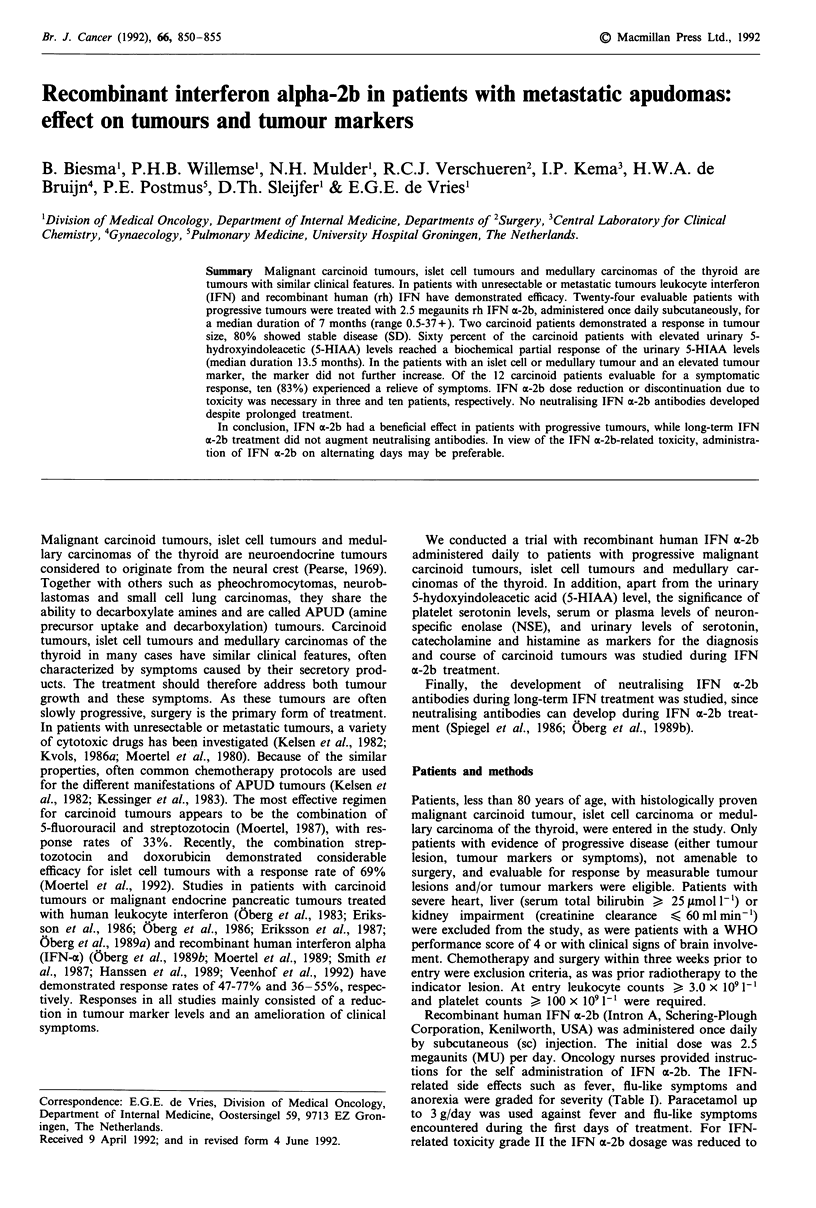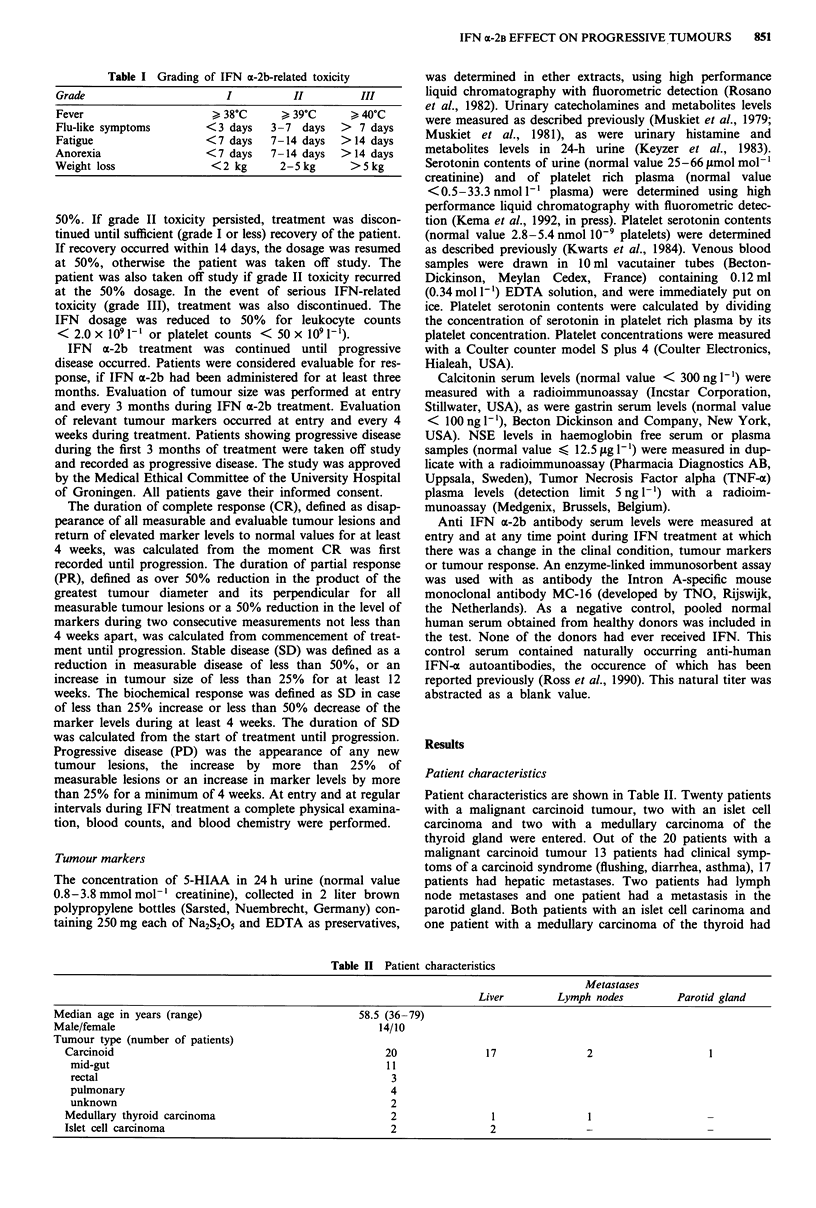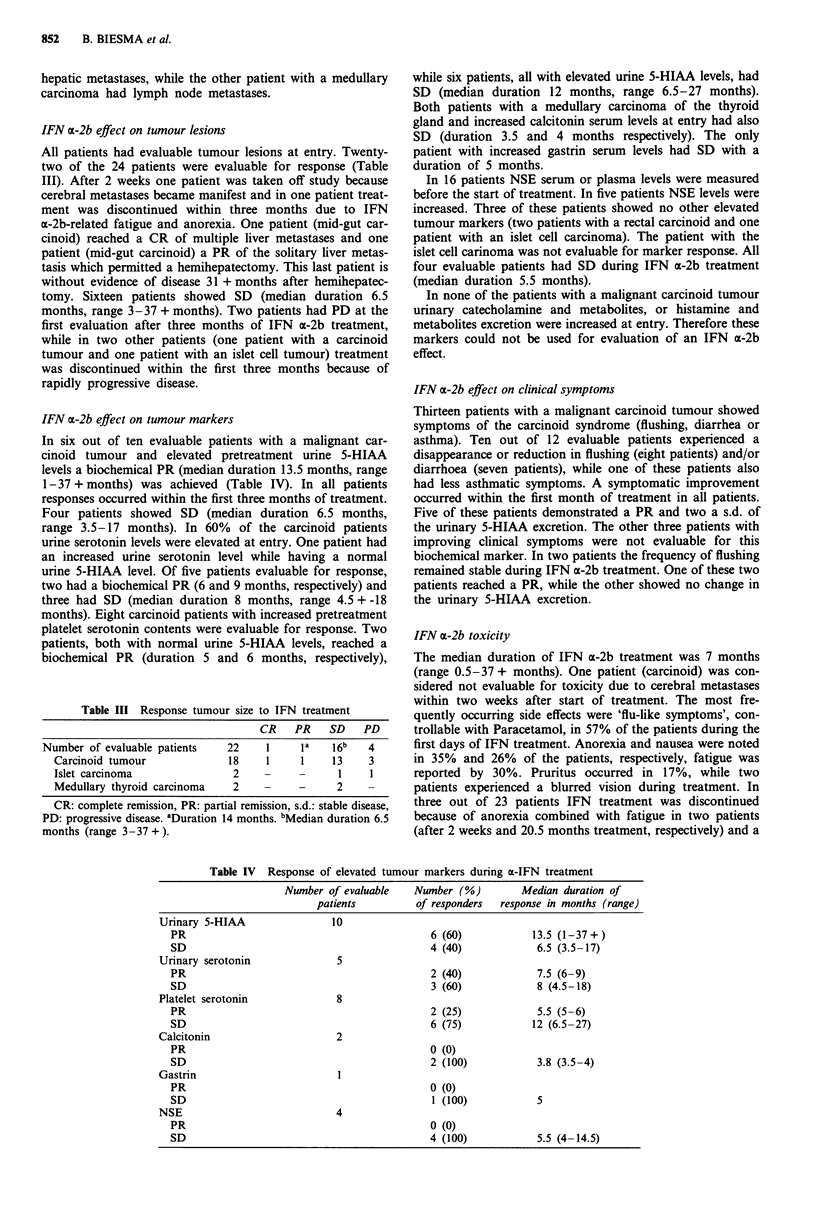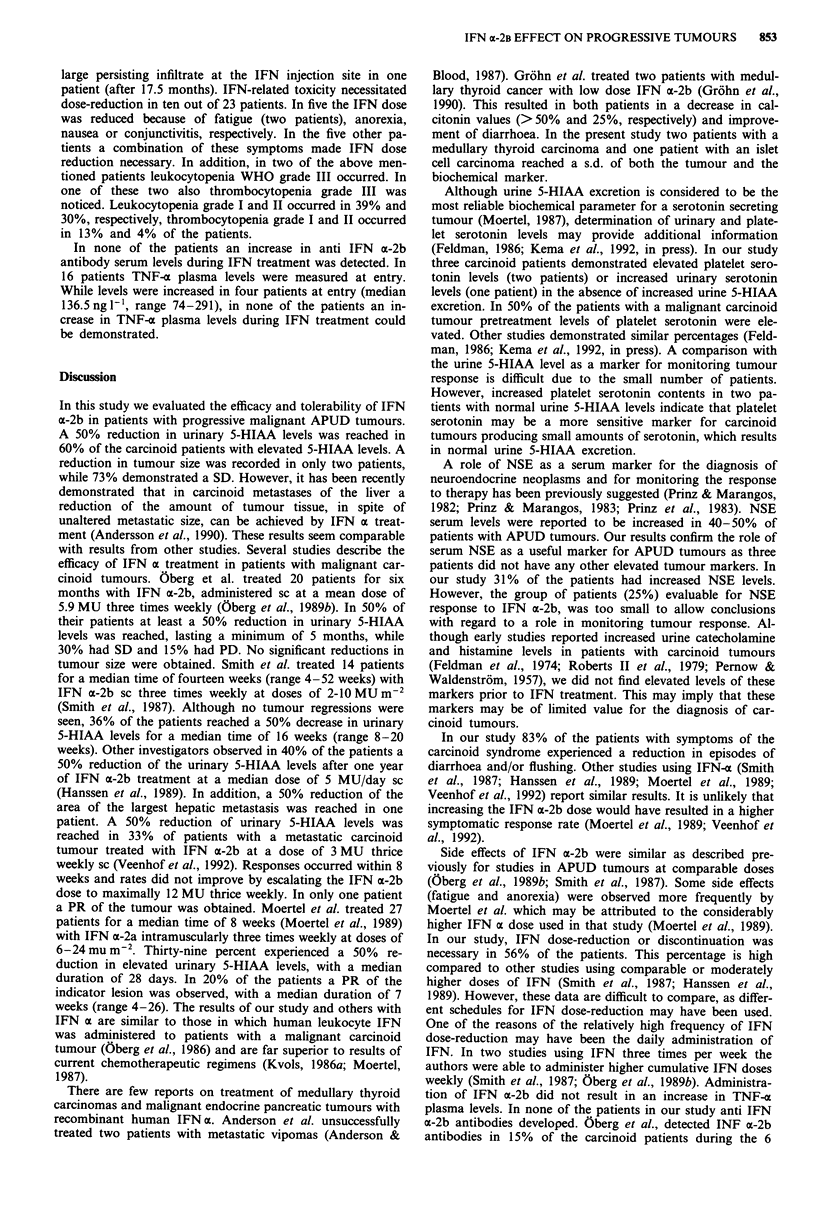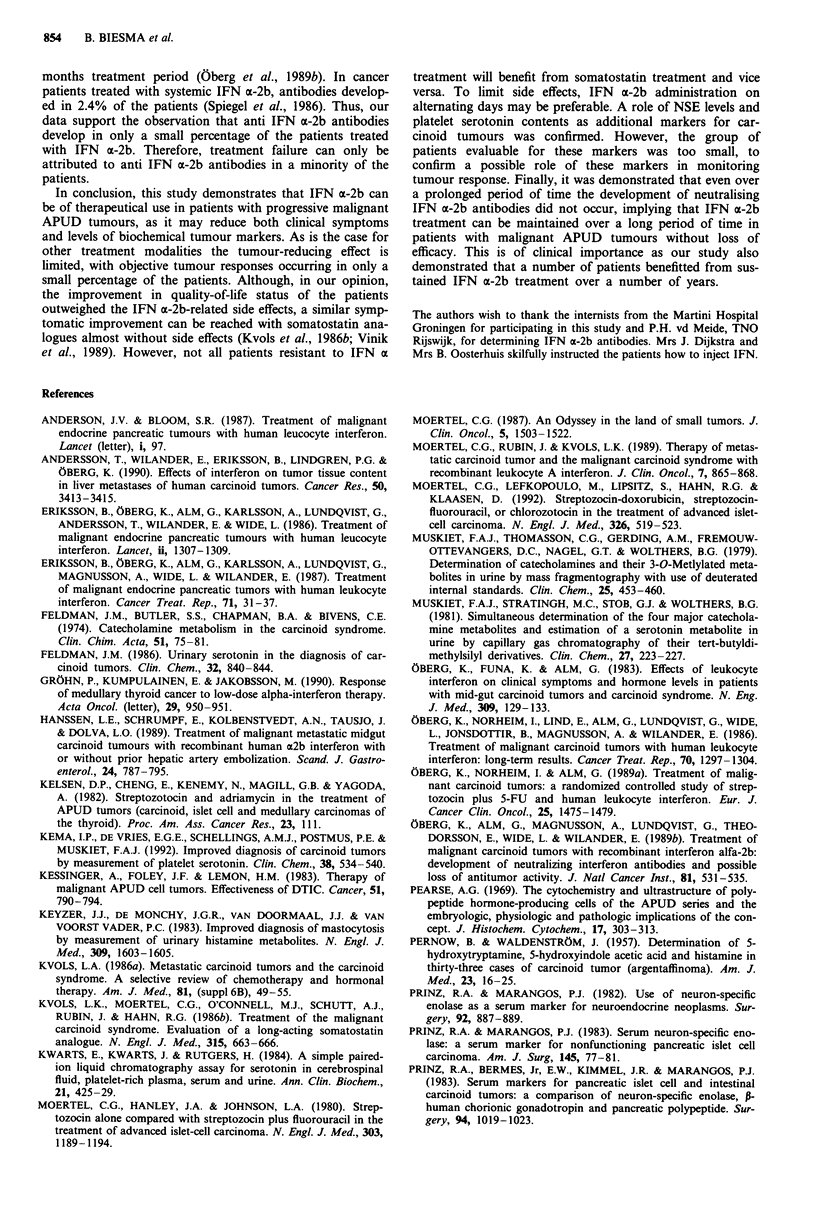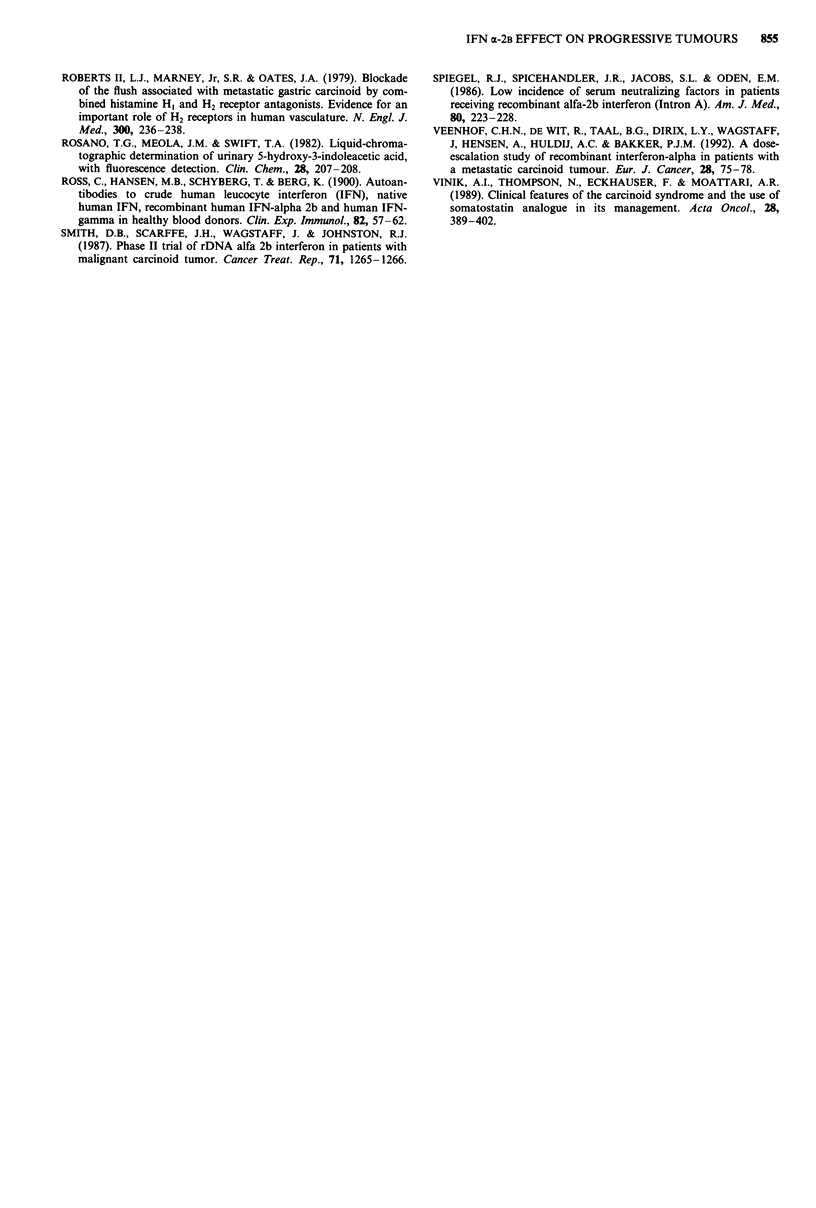# Recombinant interferon alpha-2b in patients with metastatic apudomas: effect on tumours and tumour markers.

**DOI:** 10.1038/bjc.1992.372

**Published:** 1992-11

**Authors:** B. Biesma, P. H. Willemse, N. H. Mulder, R. C. Verschueren, I. P. Kema, H. W. de Bruijn, P. E. Postmus, D. T. Sleijfer, E. G. de Vries

**Affiliations:** Department of Internal Medicine, University Hospital Groningen, The Netherlands.

## Abstract

Malignant carcinoid tumours, islet cell tumours and medullary carcinomas of the thyroid are tumours with similar clinical features. In patients with unresectable or metastatic tumours leukocyte interferon (IFN) and recombinant human (rh) IFN have demonstrated efficacy. Twenty-four evaluable patients with progressive tumours were treated with 2.5 megaunits rh IFN alpha-2b, administered once daily subcutaneously, for a median duration of 7 months (range 0.5-37+). Two carcinoid patients demonstrated a response in tumour size, 80% showed stable disease (SD). Sixty percent of the carcinoid patients with elevated urinary 5-hydroxyindoleacetic (5-HIAA) levels reached a biochemical partial response of the urinary 5-HIAA levels (median duration 13.5 months). In the patients with an islet cell or medullary tumour and an elevated tumour marker, the marker did not further increase. Of the 12 carcinoid patients evaluable for a symptomatic response, ten (83%) experienced a relieve of symptoms. IFN alpha-2b dose reduction or discontinuation due to toxicity was necessary in three and ten patients, respectively. No neutralising IFN alpha-2b antibodies developed despite prolonged treatment. In conclusion, IFN alpha-2b had a beneficial effect in patients with progressive tumours, while long-term IFN alpha-2b treatment did not augment neutralising antibodies. In view of the IFN alpha-2b-related toxicity, administration of IFN alpha-2b on alternating days may be preferable.


Br. J. Cancer (1992), 66, 850-855 © Macmillan Press Ltd., 1992

### Recombinant interferon alpha-2b in patients with metastatic apudomas: effect on tumours and tumour markers

B. Biesma[1], P.H.B. Willemse[1], N.H. Mulder[1], R.C.J. Verschueren[2], I.P. Kema[3], H.W.A. de Bruijn[4], P.E. Postmus[5], D.Th. Sleijfer[1] & E.G.E. de Vries[1]

[1]*Division of Medical Oncology, Department of Internal Medicine, Departments of* [2]*Surgery,* [3]*Central Laboratory for Clinical Chemistry,* [4]*Gynaecology,* [5]*Pulmonary Medicine, University Hospital Groningen, The Netherlands.*

**Summary** Malignant carcinoid tumours, islet cell tumours and medullary carcinomas of the thyroid are tumours with similar clinical features. In patients with unresectable or metastatic tumours leukocyte interferon (IFN) and recombinant human (rh) IFN have demonstrated efficacy. Twenty-four evaluable patients with progressive tumours were treated with 2.5 megaunits rh IFN α-2b, administered once daily subcutaneously, for a median duration of 7 months (range 0.5-37+). Two carcinoid patients demonstrated a response in tumour size, 80% showed stable disease (SD). Sixty percent of the carcinoid patients with elevated urinary 5-hydroxyindoleacetic (5-HIAA) levels reached a biochemical partial response of the urinary 5-HIAA levels (median duration 13.5 months). In the patients with an islet cell or medullary tumour and an elevated tumour marker, the marker did not further increase. Of the 12 carcinoid patients evaluable for a symptomatic response, ten (83%) experienced a relieve of symptoms. IFN α-2b dose reduction or discontinuation due to toxicity was necessary in three and ten patients, respectively. No neutralising IFN α-2b antibodies developed despite prolonged treatment.

In conclusion, IFN α-2b had a beneficial effect in patients with progressive tumours, while long-term IFN α-2b treatment did not augment neutralising antibodies. In view of the IFN α-2b-related toxicity, administration of IFN α-2b on alternating days may be preferable.

Malignant carcinoid tumours, islet cell tumours and medullary carcinomas of the thyroid are neuroendocrine tumours considered to originate from the neural crest (Pearse, 1969). Together with others such as pheochromocytomas, neuroblastomas and small cell lung carcinomas, they share the ability to decarboxylate amines and are called APUD (amine precursor uptake and decarboxylation) tumours. Carcinoid tumours, islet cell tumours and medullary carcinomas of the thyroid in many cases have similar clinical features, often characterized by symptoms caused by their secretory products. The treatment should therefore address both tumour growth and these symptoms. As these tumours are often slowly progressive, surgery is the primary form of treatment. In patients with unresectable or metastatic tumours, a variety of cytotoxic drugs has been investigated (Kelsen *et al.*, 1982; Kvols, 1986*a*; Moertel *et al.*, 1980). Because of the similar properties, often common chemotherapy protocols are used for the different manifestations of APUD tumours (Kelsen *et al.*, 1982; Kessinger *et al.*, 1983). The most effective regimen for carcinoid tumours appears to be the combination of 5-fluorouracil and streptozotocin (Moertel, 1987), with response rates of 33%. Recently, the combination streptozotocin and doxorubicin demonstrated considerable efficacy for islet cell tumours with a response rate of 69% (Moertel *et al.*, 1992). Studies in patients with carcinoid tumours or malignant endocrine pancreatic tumours treated with human leukocyte interferon (Öberg *et al.*, 1983; Eriksson *et al.*, 1986; Öberg *et al.*, 1986; Eriksson *et al.*, 1987; Öberg *et al.*, 1989*a*) and recombinant human interferon alpha (IFN-α) (Öberg *et al.*, 1989*b*; Moertel *et al.*, 1989; Smith *et al.*, 1987; Hanssen *et al.*, 1989; Veenhof *et al.*, 1992) have demonstrated response rates of 47-77% and 36-55%, respectively. Responses in all studies mainly consisted of a reduction in tumour marker levels and an amelioration of clinical symptoms.

Correspondence: E.G.E. de Vries, Division of Medical Oncology, Department of Internal Medicine, Oostersingel 59, 9713 EZ Groningen, The Netherlands.
Received 9 April 1992; and in revised form 4 June 1992.

We conducted a trial with recombinant human IFN α-2b administered daily to patients with progressive malignant carcinoid tumours, islet cell tumours and medullary carcinomas of the thyroid. In addition, apart from the urinary 5-hydoxyindoleacetic acid (5-HIAA) level, the significance of platelet serotonin levels, serum or plasma levels of neuron-specific enolase (NSE), and urinary levels of serotonin, catecholamine and histamine as markers for the diagnosis and course of carcinoid tumours was studied during IFN α-2b treatment.

Finally, the development of neutralising IFN α-2b antibodies during long-term IFN treatment was studied, since neutralising antibodies can develop during IFN α-2b treatment (Spiegel *et al.*, 1986; Öberg *et al.*, 1989b).

### Patients and methods

Patients, less than 80 years of age, with histologically proven malignant carcinoid tumour, islet cell carcinoma or medullary carcinoma of the thyroid, were entered in the study. Only patients with evidence of progressive disease (either tumour lesion, tumour markers or symptoms), not amenable to surgery, and evaluable for response by measurable tumour lesions and/or tumour markers were eligible. Patients with severe heart, liver (serum total bilirubin $\geqslant 25\,\mu mol\,l^{-1}$) or kidney impairment (creatinine clearance $\leqslant 60\,ml\,min^{-1}$) were excluded from the study, as were patients with a WHO performance score of 4 or with clinical signs of brain involvement. Chemotherapy and surgery within three weeks prior to entry were exclusion criteria, as was prior radiotherapy to the indicator lesion. At entry leukocyte counts $\geqslant 3.0 \times 10^9\,l^{-1}$ and platelet counts $\geqslant 100 \times 10^9\,l^{-1}$ were required.

Recombinant human IFN α-2b (Intron A, Schering-Plough Corporation, Kenilworth, USA) was administered once daily by subcutaneous (sc) injection. The initial dose was 2.5 megaunits (MU) per day. Oncology nurses provided instructions for the self administration of IFN α-2b. The IFN-related side effects such as fever, flu-like symptoms and anorexia were graded for severity (Table I). Paracetamol up to 3 g/day was used against fever and flu-like symptoms encountered during the first days of treatment. For IFN-related toxicity grade II the IFN α-2b dosage was reduced to

**Table I** Grading of IFN α-2b-related toxicity

| *Grade* | *I* | *II* | *III* |
|---|---|---|---|
| Fever | $\geq$ 38°C | $\geq$ 39°C | $\geq$ 40°C |
| Flu-like symptoms | $<$3 days | 3–7 days | $>$ 7 days |
| Fatigue | $<$7 days | 7–14 days | $>$14 days |
| Anorexia | $<$7 days | 7–14 days | $>$14 days |
| Weight loss | $<$2 kg | 2–5 kg | $>$5 kg |

50%. If grade II toxicity persisted, treatment was discontinued until sufficient (grade I or less) recovery of the patient. If recovery occurred within 14 days, the dosage was resumed at 50%, otherwise the patient was taken off study. The patient was also taken off study if grade II toxicity recurred at the 50% dosage. In the event of serious IFN-related toxicity (grade III), treatment was also discontinued. The IFN dosage was reduced to 50% for leukocyte counts $< 2.0 \times 10^9 l^{-1}$ or platelet counts $< 50 \times 10^9 l^{-1}$).

IFN α-2b treatment was continued until progressive disease occurred. Patients were considered evaluable for response, if IFN α-2b had been administered for at least three months. Evaluation of tumour size was performed at entry and every 3 months during IFN α-2b treatment. Evaluation of relevant tumour markers occurred at entry and every 4 weeks during treatment. Patients showing progressive disease during the first 3 months of treatment were taken off study and recorded as progressive disease. The study was approved by the Medical Ethical Committee of the University Hospital of Groningen. All patients gave their informed consent.

The duration of complete response (CR), defined as disappearance of all measurable and evaluable tumour lesions and return of elevated marker levels to normal values for at least 4 weeks, was calculated from the moment CR was first recorded until progression. The duration of partial response (PR), defined as over 50% reduction in the product of the greatest tumour diameter and its perpendicular for all measurable tumour lesions or a 50% reduction in the level of markers during two consecutive measurements not less than 4 weeks apart, was calculated from commencement of treatment until progression. Stable disease (SD) was defined as a reduction in measurable disease of less than 50%, or an increase in tumour size of less than 25% for at least 12 weeks. The biochemical response was defined as SD in case of less than 25% increase or less than 50% decrease of the marker levels during at least 4 weeks. The duration of SD was calculated from the start of treatment until progression. Progressive disease (PD) was the appearance of any new tumour lesions, the increase by more than 25% of measurable lesions or an increase in marker levels by more than 25% for a minimum of 4 weeks. At entry and at regular intervals during IFN treatment a complete physical examination, blood counts, and blood chemistry were performed.

### *Tumour markers*

The concentration of 5-HIAA in 24 h urine (normal value 0.8–3.8 mmol mol$^{-1}$ creatinine), collected in 2 liter brown polypropylene bottles (Sarsted, Nuembrecht, Germany) containing 250 mg each of $Na_2S_2O_5$ and EDTA as preservatives, was determined in ether extracts, using high performance liquid chromatography with fluorometric detection (Rosano *et al.*, 1982). Urinary catecholamines and metabolites levels were measured as described previously (Muskiet *et al.*, 1979; Muskiet *et al.*, 1981), as were urinary histamine and metabolites levels in 24-h urine (Keyzer *et al.*, 1983). Serotonin contents of urine (normal value 25–66 μmol mol$^{-1}$ creatinine) and of platelet rich plasma (normal value $<$0.5–33.3 nmol l$^{-1}$ plasma) were determined using high performance liquid chromatography with fluorometric detection (Kema *et al.*, 1992, in press). Platelet serotonin contents (normal value 2.8–5.4 nmol 10$^{-9}$ platelets) were determined as described previously (Kwarts *et al.*, 1984). Venous blood samples were drawn in 10 ml vacutainer tubes (Becton-Dickinson, Meylan Cedex, France) containing 0.12 ml (0.34 mol l$^{-1}$) EDTA solution, and were immediately put on ice. Platelet serotonin contents were calculated by dividing the concentration of serotonin in platelet rich plasma by its platelet concentration. Platelet concentrations were measured with a Coulter counter model S plus 4 (Coulter Electronics, Hialeah, USA).

Calcitonin serum levels (normal value $<$ 300 ng l$^{-1}$) were measured with a radioimmunoassay (Incstar Corporation, Stillwater, USA), as were gastrin serum levels (normal value $<$ 100 ng l$^{-1}$), Becton Dickinson and Company, New York, USA). NSE levels in haemoglobin free serum or plasma samples (normal value $\leq$ 12.5 μg l$^{-1}$) were measured in duplicate with a radioimmunoassay (Pharmacia Diagnostics AB, Uppsala, Sweden), Tumor Necrosis Factor alpha (TNF-α) plasma levels (detection limit 5 ng l$^{-1}$) with a radioimmunoassay (Medgenix, Brussels, Belgium).

Anti IFN α-2b antibody serum levels were measured at entry and at any time point during IFN treatment at which there was a change in the clinal condition, tumour markers or tumour response. An enzyme-linked immunosorbent assay was used with as antibody the Intron A-specific mouse monoclonal antibody MC-16 (developed by TNO, Rijswijk, the Netherlands). As a negative control, pooled normal human serum obtained from healthy donors was included in the test. None of the donors had ever received IFN. This control serum contained naturally occurring anti-human IFN-α autoantibodies, the occurence of which has been reported previously (Ross *et al.*, 1990). This natural titer was abstracted as a blank value.

### Results

### *Patient characteristics*

Patient characteristics are shown in Table II. Twenty patients with a malignant carcinoid tumour, two with an islet cell carcinoma and two with a medullary carcinoma of the thyroid gland were entered. Out of the 20 patients with a malignant carcinoid tumour 13 patients had clinical symptoms of a carcinoid syndrome (flushing, diarrhea, asthma), 17 patients had hepatic metastases. Two patients had lymph node metastases and one patient had a metastasis in the parotid gland. Both patients with an islet cell carinoma and one patient with a medullary carcinoma of the thyroid had

**Table II** Patient characteristics

| | | *Metastases* | | |
|---|---|---|---|---|
| | | *Liver* | *Lymph nodes* | *Parotid gland* |
| Median age in years (range) | 58.5 (36–79) | | | |
| Male/female | 14/10 | | | |
| Tumour type (number of patients) | | | | |
| Carcinoid | 20 | 17 | 2 | 1 |
| mid-gut | 11 | | | |
| rectal | 3 | | | |
| pulmonary | 4 | | | |
| unknown | 2 | | | |
| Medullary thyroid carcinoma | 2 | 1 | 1 | – |
| Islet cell carcinoma | 2 | 2 | – | – |

hepatic metastases, while the other patient with a medullary carcinoma had lymph node metastases.

*IFN α-2b effect on tumour lesions*

All patients had evaluable tumour lesions at entry. Twenty-two of the 24 patients were evaluable for response (Table III). After 2 weeks one patient was taken off study because cerebral metastases became manifest and in one patient treatment was discontinued within three months due to IFN α-2b-related fatigue and anorexia. One patient (mid-gut carcinoid) reached a CR of multiple liver metastases and one patient (mid-gut carcinoid) a PR of the solitary liver metastasis which permitted a hemihepatectomy. This last patient is without evidence of disease 31 + months after hemihepatectomy. Sixteen patients showed SD (median duration 6.5 months, range 3–37 + months). Two patients had PD at the first evaluation after three months of IFN α-2b treatment, while in two other patients (one patient with a carcinoid tumour and one patient with an islet cell tumour) treatment was discontinued within the first three months because of rapidly progressive disease.

*IFN α-2b effect on tumour markers*

In six out of ten evaluable patients with a malignant carcinoid tumour and elevated pretreatment urine 5-HIAA levels a biochemical PR (median duration 13.5 months, range 1–37 + months) was achieved (Table IV). In all patients responses occurred within the first three months of treatment. Four patients showed SD (median duration 6.5 months, range 3.5–17 months). In 60% of the carcinoid patients urine serotonin levels were elevated at entry. One patient had an increased urine serotonin level while having a normal urine 5-HIAA level. Of five patients evaluable for response, two had a biochemical PR (6 and 9 months, respectively) and three had SD (median duration 8 months, range 4.5 + -18 months). Eight carcinoid patients with increased pretreatment platelet serotonin contents were evaluable for response. Two patients, both with normal urine 5-HIAA levels, reached a biochemical PR (duration 5 and 6 months, respectively), while six patients, all with elevated urine 5-HIAA levels, had SD (median duration 12 months, range 6.5–27 months). Both patients with a medullary carcinoma of the thyroid gland and increased calcitonin serum levels at entry had also SD (duration 3.5 and 4 months respectively). The only patient with increased gastrin serum levels had SD with a duration of 5 months.

In 16 patients NSE serum or plasma levels were measured before the start of treatment. In five patients NSE levels were increased. Three of these patients showed no other elevated tumour markers (two patients with a rectal carcinoid and one patient with an islet cell carcinoma). The patient with the islet cell carinoma was not evaluable for marker response. All four evaluable patients had SD during IFN α-2b treatment (median duration 5.5 months).

In none of the patients with a malignant carcinoid tumour urinary catecholamine and metabolites, or histamine and metabolites excretion were increased at entry. Therefore these markers could not be used for evaluation of an IFN α-2b effect.

**Table III** Response tumour size to IFN treatment

| | | *CR* | *PR* | *SD* | *PD* |
|---|---|---|---|---|---|
| Number of evaluable patients | 22 | 1 | 1[a] | 16[b] | 4 |
| Carcinoid tumour | 18 | 1 | 1 | 13 | 3 |
| Islet carcinoma | 2 | – | – | 1 | 1 |
| Medullary thyroid carcinoma | 2 | – | – | 2 | – |

CR: complete remission, PR: partial remission, s.d.: stable disease, PD: progressive disease. [a]Duration 14 months. [b]Median duration 6.5 months (range 3–37 +).

*IFN α-2b effect on clinical symptoms*

Thirteen patients with a malignant carcinoid tumour showed symptoms of the carcinoid syndrome (flushing, diarrhea or asthma). Ten out of 12 evaluable patients experienced a disappearance or reduction in flushing (eight patients) and/or diarrhoea (seven patients), while one of these patients also had less asthmatic symptoms. A symptomatic improvement occurred within the first month of treatment in all patients. Five of these patients demonstrated a PR and two a s.d. of the urinary 5-HIAA excretion. The other three patients with improving clinical symptoms were not evaluable for this biochemical marker. In two patients the frequency of flushing remained stable during IFN α-2b treatment. One of these two patients reached a PR, while the other showed no change in the urinary 5-HIAA excretion.

*IFN α-2b toxicity*

The median duration of IFN α-2b treatment was 7 months (range 0.5–37 + months). One patient (carcinoid) was considered not evaluable for toxicity due to cerebral metastases within two weeks after start of treatment. The most frequently occurring side effects were 'flu-like symptoms', controllable with Paracetamol, in 57% of the patients during the first days of IFN treatment. Anorexia and nausea were noted in 35% and 26% of the patients, respectively, fatigue was reported by 30%. Pruritus occurred in 17%, while two patients experienced a blurred vision during treatment. In three out of 23 patients IFN treatment was discontinued because of anorexia combined with fatigue in two patients (after 2 weeks and 20.5 months treatment, respectively) and a

**Table IV** Response of elevated tumour markers during α-IFN treatment

| | *Number of evaluable patients* | *Number (%) of responders* | *Median duration of response in months (range)* |
|---|---|---|---|
| Urinary 5-HIAA | 10 | | |
| PR | | 6 (60) | 13.5 (1–37 +) |
| SD | | 4 (40) | 6.5 (3.5–17) |
| Urinary serotonin | 5 | | |
| PR | | 2 (40) | 7.5 (6–9) |
| SD | | 3 (60) | 8 (4.5–18) |
| Platelet serotonin | 8 | | |
| PR | | 2 (25) | 5.5 (5–6) |
| SD | | 6 (75) | 12 (6.5–27) |
| Calcitonin | 2 | | |
| PR | | 0 (0) | |
| SD | | 2 (100) | 3.8 (3.5–4) |
| Gastrin | 1 | | |
| PR | | 0 (0) | |
| SD | | 1 (100) | 5 |
| NSE | 4 | | |
| PR | | 0 (0) | |
| SD | | 4 (100) | 5.5 (4–14.5) |

large persisting infiltrate at the IFN injection site in one patient (after 17.5 months). IFN-related toxicity necessitated dose-reduction in ten out of 23 patients. In five the IFN dose was reduced because of fatigue (two patients), anorexia, nausea or conjunctivitis, respectively. In the five other patients a combination of these symptoms made IFN dose reduction necessary. In addition, in two of the above mentioned patients leukocytopenia WHO grade III occurred. In one of these two also thrombocytopenia grade III was noticed. Leukocytopenia grade I and II occurred in 39% and 30%, respectively, thrombocytopenia grade I and II occurred in 13% and 4% of the patients.

In none of the patients an increase in anti IFN α-2b antibody serum levels during IFN treatment was detected. In 16 patients TNF-α plasma levels were measured at entry. While levels were increased in four patients at entry (median 136.5 ng $l^{-1}$, range 74–291), in none of the patients an increase in TNF-α plasma levels during IFN treatment could be demonstrated.

### Discussion

In this study we evaluated the efficacy and tolerability of IFN α-2b in patients with progressive malignant APUD tumours. A 50% reduction in urinary 5-HIAA levels was reached in 60% of the carcinoid patients with elevated 5-HIAA levels. A reduction in tumour size was recorded in only two patients, while 73% demonstrated a SD. However, it has been recently demonstrated that in carcinoid metastases of the liver a reduction of the amount of tumour tissue, in spite of unaltered metastatic size, can be achieved by IFN α treatment (Andersson *et al.*, 1990). These results seem comparable with results from other studies. Several studies describe the efficacy of IFN α treatment in patients with malignant carcinoid tumours. Öberg et al. treated 20 patients for six months with IFN α-2b, administered sc at a mean dose of 5.9 MU three times weekly (Öberg *et al.*, 1989*b*). In 50% of their patients at least a 50% reduction in urinary 5-HIAA levels was reached, lasting a minimum of 5 months, while 30% had SD and 15% had PD. No significant reductions in tumour size were obtained. Smith *et al.* treated 14 patients for a median time of fourteen weeks (range 4–52 weeks) with IFN α-2b sc three times weekly at doses of 2-10 MU $m^{-2}$ (Smith *et al.*, 1987). Although no tumour regressions were seen, 36% of the patients reached a 50% decrease in urinary 5-HIAA levels for a median time of 16 weeks (range 8–20 weeks). Other investigators observed in 40% of the patients a 50% reduction of the urinary 5-HIAA levels after one year of IFN α-2b treatment at a median dose of 5 MU/day sc (Hanssen *et al.*, 1989). In addition, a 50% reduction of the area of the largest hepatic metastasis was reached in one patient. A 50% reduction of urinary 5-HIAA levels was reached in 33% of patients with a metastatic carcinoid tumour treated with IFN α-2b at a dose of 3 MU thrice weekly sc (Veenhof *et al.*, 1992). Responses occurred within 8 weeks and rates did not improve by escalating the IFN α-2b dose to maximally 12 MU thrice weekly. In only one patient a PR of the tumour was obtained. Moertel *et al.* treated 27 patients for a median time of 8 weeks (Moertel *et al.*, 1989) with IFN α-2a intramuscularly three times weekly at doses of 6–24 mu $m^{-2}$. Thirty-nine percent experienced a 50% reduction in elevated urinary 5-HIAA levels, with a median duration of 28 days. In 20% of the patients a PR of the indicator lesion was observed, with a median duration of 7 weeks (range 4–26). The results of our study and others with IFN α are similar to those in which human leukocyte IFN was administered to patients with a malignant carcinoid tumour (Öberg *et al.*, 1986) and are far superior to results of current chemotherapeutic regimens (Kvols, 1986*a*; Moertel, 1987).

There are few reports on treatment of medullary thyroid carcinomas and malignant endocrine pancreatic tumours with recombinant human IFN α. Anderson *et al.* unsuccessfully treated two patients with metastatic vipomas (Anderson & Blood, 1987). Gröhn *et al.* treated two patients with medullary thyroid cancer with low dose IFN α-2b (Gröhn *et al.*, 1990). This resulted in both patients in a decrease in calcitonin values (>50% and 25%, respectively) and improvement of diarrhoea. In the present study two patients with a medullary thyroid carcinoma and one patient with an islet cell carcinoma reached a s.d. of both the tumour and the biochemical marker.

Although urine 5-HIAA excretion is considered to be the most reliable biochemical parameter for a serotonin secreting tumour (Moertel, 1987), determination of urinary and platelet serotonin levels may provide additional information (Feldman, 1986; Kema *et al.*, 1992, in press). In our study three carcinoid patients demonstrated elevated platelet serotonin levels (two patients) or increased urinary serotonin levels (one patient) in the absence of increased urine 5-HIAA excretion. In 50% of the patients with a malignant carcinoid tumour pretreatment levels of platelet serotonin were elevated. Other studies demonstrated similar percentages (Feldman, 1986; Kema *et al.*, 1992, in press). A comparison with the urine 5-HIAA level as a marker for monitoring tumour response is difficult due to the small number of patients. However, increased platelet serotonin contents in two patients with normal urine 5-HIAA levels indicate that platelet serotonin may be a more sensitive marker for carcinoid tumours producing small amounts of serotonin, which results in normal urine 5-HIAA excretion.

A role of NSE as a serum marker for the diagnosis of neuroendocrine neoplasms and for monitoring the response to therapy has been previously suggested (Prinz & Marangos, 1982; Prinz & Marangos, 1983; Prinz *et al.*, 1983). NSE serum levels were reported to be increased in 40–50% of patients with APUD tumours. Our results confirm the role of serum NSE as a useful marker for APUD tumours as three patients did not have any other elevated tumour markers. In our study 31% of the patients had increased NSE levels. However, the group of patients (25%) evaluable for NSE response to IFN α-2b, was too small to allow conclusions with regard to a role in monitoring tumour response. Although early studies reported increased urine catecholamine and histamine levels in patients with carcinoid tumours (Feldman *et al.*, 1974; Roberts II *et al.*, 1979; Pernow & Waldenström, 1957), we did not find elevated levels of these markers prior to IFN treatment. This may imply that these markers may be of limited value for the diagnosis of carcinoid tumours.

In our study 83% of the patients with symptoms of the carcinoid syndrome experienced a reduction in episodes of diarrhoea and/or flushing. Other studies using IFN-α (Smith *et al.*, 1987; Hanssen *et al.*, 1989; Moertel *et al.*, 1989; Veenhof *et al.*, 1992) report similar results. It is unlikely that increasing the IFN α-2b dose would have resulted in a higher symptomatic response rate (Moertel *et al.*, 1989; Veenhof *et al.*, 1992).

Side effects of IFN α-2b were similar as described previously for studies in APUD tumours at comparable doses (Öberg *et al.*, 1989*b*; Smith *et al.*, 1987). Some side effects (fatigue and anorexia) were observed more frequently by Moertel *et al.* which may be attributed to the considerably higher IFN α dose used in that study (Moertel *et al.*, 1989). In our study, IFN dose-reduction or discontinuation was necessary in 56% of the patients. This percentage is high compared to other studies using comparable or moderately higher doses of IFN (Smith *et al.*, 1987; Hanssen *et al.*, 1989). However, these data are difficult to compare, as different schedules for IFN dose-reduction may have been used. One of the reasons of the relatively high frequency of IFN dose-reduction may have been the daily administration of IFN. In two studies using IFN three times per week the authors were able to administer higher cumulative IFN doses weekly (Smith *et al.*, 1987; Öberg *et al.*, 1989*b*). Administration of IFN α-2b did not result in an increase in TNF-α plasma levels. In none of the patients in our study anti IFN α-2b antibodies developed. Öberg *et al.*, detected INF α-2b antibodies in 15% of the carcinoid patients during the 6

months treatment period (Öberg *et al.*, 1989*b*). In cancer patients treated with systemic IFN α-2b, antibodies developed in 2.4% of the patients (Spiegel *et al.*, 1986). Thus, our data support the observation that anti IFN α-2b antibodies develop in only a small percentage of the patients treated with IFN α-2b. Therefore, treatment failure can only be attributed to anti IFN α-2b antibodies in a minority of the patients.

In conclusion, this study demonstrates that IFN α-2b can be of therapeutical use in patients with progressive malignant APUD tumours, as it may reduce both clinical symptoms and levels of biochemical tumour markers. As is the case for other treatment modalities the tumour-reducing effect is limited, with objective tumour responses occurring in only a small percentage of the patients. Although, in our opinion, the improvement in quality-of-life status of the patients outweighed the IFN α-2b-related side effects, a similar symptomatic improvement can be reached with somatostatin analogues almost without side effects (Kvols *et al.*, 1986*b*; Vinik *et al.*, 1989). However, not all patients resistant to IFN α treatment will benefit from somatostatin treatment and vice versa. To limit side effects, IFN α-2b administration on alternating days may be preferable. A role of NSE levels and platelet serotonin contents as additional markers for carcinoid tumours was confirmed. However, the group of patients evaluable for these markers was too small, to confirm a possible role of these markers in monitoring tumour response. Finally, it was demonstrated that even over a prolonged period of time the development of neutralising IFN α-2b antibodies did not occur, implying that IFN α-2b treatment can be maintained over a long period of time in patients with malignant APUD tumours without loss of efficacy. This is of clinical importance as our study also demonstrated that a number of patients benefitted from sustained IFN α-2b treatment over a number of years.

The authors wish to thank the internists from the Martini Hospital Groningen for participating in this study and P.H. vd Meide, TNO Rijswijk, for determining IFN α-2b antibodies. Mrs J. Dijkstra and Mrs B. Oosterhuis skilfully instructed the patients how to inject IFN.

### References

ANDERSON, J.V. & BLOOM, S.R. (1987). Treatment of malignant endocrine pancreatic tumours with human leucocyte interferon. *Lancet* (letter), **i**, 97.

ANDERSSON, T., WILANDER, E., ERIKSSON, B., LINDGREN, P.G. & ÖBERG, K. (1990). Effects of interferon on tumor tissue content in liver metastases of human carcinoid tumors. *Cancer Res.*, **50**, 3413–3415.

ERIKSSON, B., ÖBERG, K., ALM, G., KARLSSON, A., LUNDQVIST, G., ANDERSSON, T., WILANDER, E. & WIDE, L. (1986). Treatment of malignant endocrine pancreatic tumours with human leucocyte interferon. *Lancet*, **ii**, 1307–1309.

ERIKSSON, B., ÖBERG, K., ALM, G., KARLSSON, A., LUNDQVIST, G., MAGNUSSON, A., WIDE, L. & WILANDER, E. (1987). Treatment of malignant endocrine pancreatic tumors with human leukocyte interferon. *Cancer Treat. Rep.*, **71**, 31–37.

FELDMAN, J.M., BUTLER, S.S., CHAPMAN, B.A. & BIVENS, C.E. (1974). Catecholamine metabolism in the carcinoid syndrome. *Clin. Chim. Acta*, **51**, 75–81.

FELDMAN, J.M. (1986). Urinary serotonin in the diagnosis of carcinoid tumors. *Clin. Chem.*, **32**, 840–844.

GRÖHN, P., KUMPULAINEN, E. & JAKOBSSON, M. (1990). Response of medullary thyroid cancer to low-dose alpha-interferon therapy. *Acta Oncol.* (letter), **29**, 950–951.

HANSSEN, L.E., SCHRUMPF, E., KOLBENSTVEDT, A.N., TAUSJO, J. & DOLVA, L.O. (1989). Treatment of malignant metastatic midgut carcinoid tumours with recombinant human α2b interferon with or without prior hepatic artery embolization. *Scand. J. Gastroenterol.*, **24**, 787–795.

KELSEN, D.P., CHENG, E., KENEMY, N., MAGILL, G.B. & YAGODA, A. (1982). Streptozotocin and adriamycin in the treatment of APUD tumors (carcinoid, islet cell and medullary carcinomas of the thyroid). *Proc. Am. Ass. Cancer Res.*, **23**, 111.

KEMA, I.P., DE VRIES, E.G.E., SCHELLINGS, A.M.J., POSTMUS, P.E. & MUSKIET, F.A.J. (1992). Improved diagnosis of carcinoid tumors by measurement of platelet serotonin. *Clin. Chem.*, **38**, 534–540.

KESSINGER, A., FOLEY, J.F. & LEMON, H.M. (1983). Therapy of malignant APUD cell tumors. Effectiveness of DTIC. *Cancer*, **51**, 790–794.

KEYZER, J.J., DE MONCHY, J.G.R., VAN DOORMAAL, J.J. & VAN VOORST VADER, P.C. (1983). Improved diagnosis of mastocytosis by measurement of urinary histamine metabolites. *N. Engl. J. Med.*, **309**, 1603–1605.

KVOLS, L.A. (1986*a*). Metastatic carcinoid tumors and the carcinoid syndrome. A selective review of chemotherapy and hormonal therapy. *Am. J. Med.*, **81**, (suppl 6B), 49–55.

KVOLS, L.K., MOERTEL, C.G., O'CONNELL, M.J., SCHUTT, A.J., RUBIN, J. & HAHN, R.G. (1986*b*). Treatment of the malignant carcinoid syndrome. Evaluation of a long-acting somatostatin analogue. *N. Engl. J. Med.*, **315**, 663–666.

KWARTS, E., KWARTS, J. & RUTGERS, H. (1984). A simple paired-ion liquid chromatography assay for serotonin in cerebrospinal fluid, platelet-rich plasma, serum and urine. *Ann. Clin. Biochem.*, **21**, 425–29.

MOERTEL, C.G., HANLEY, J.A. & JOHNSON, L.A. (1980). Streptozocin alone compared with streptozocin plus fluorouracil in the treatment of advanced islet-cell carcinoma. *N. Engl. J. Med.*, **303**, 1189–1194.

MOERTEL, C.G. (1987). An Odyssey in the land of small tumors. *J. Clin. Oncol.*, **5**, 1503–1522.

MOERTEL, C.G., RUBIN, J. & KVOLS, L.K. (1989). Therapy of metastatic carcinoid tumor and the malignant carcinoid syndrome with recombinant leukocyte A interferon. *J. Clin. Oncol.*, **7**, 865–868.

MOERTEL, C.G., LEFKOPOULO, M., LIPSITZ, S., HAHN, R.G. & KLAASEN, D. (1992). Streptozocin-doxorubicin, streptozocin-fluorouracil, or chlorozotocin in the treatment of advanced islet-cell carcinoma. *N. Engl. J. Med.*, **326**, 519–523.

MUSKIET, F.A.J., THOMASSON, C.G., GERDING, A.M., FREMOUW-OTTEVANGERS, D.C., NAGEL, G.T. & WOLTHERS, B.G. (1979). Determination of catecholamines and their 3-*O*-Metlylated metabolites in urine by mass fragmentography with use of deuterated internal standards. *Clin. Chem.*, **25**, 453–460.

MUSKIET, F.A.J., STRATINGH, M.C., STOB, G.J. & WOLTHERS, B.G. (1981). Simultaneous determination of the four major catecholamine metabolites and estimation of a serotonin metabolite in urine by capillary gas chromatography of their tert-butyldimethylsilyl derivatives. *Clin. Chem.*, **27**, 223–227.

ÖBERG, K., FUNA, K. & ALM, G. (1983). Effects of leukocyte interferon on clinical symptoms and hormone levels in patients with mid-gut carcinoid tumors and carcinoid syndrome. *N. Eng. J. Med.*, **309**, 129–133.

ÖBERG, K., NORHEIM, I., LIND, E., ALM, G., LUNDQVIST, G., WIDE, L., JONSDOTTIR, B., MAGNUSSON, A. & WILANDER, E. (1986). Treatment of malignant carcinoid tumors with human leukocyte interferon: long-term results. *Cancer Treat. Rep.*, **70**, 1297–1304.

ÖBERG, K., NORHEIM, I. & ALM, G. (1989*a*). Treatment of malignant carcinoid tumors: a randomized controlled study of streptozocin plus 5-FU and human leukocyte interferon. *Eur. J. Cancer Clin. Oncol.*, **25**, 1475–1479.

ÖBERG, K., ALM, G., MAGNUSSON, A., LUNDQVIST, G., THEODORSSON, E., WIDE, L. & WILANDER, E. (1989*b*). Treatment of malignant carcinoid tumors with recombinant interferon alfa-2b: development of neutralizing interferon antibodies and possible loss of antitumor activity. *J. Natl Cancer Inst.*, **81**, 531–535.

PEARSE, A.G. (1969). The cytochemistry and ultrastructure of polypeptide hormone-producing cells of the APUD series and the embryologic, physiologic and pathologic implications of the concept. *J. Histochem. Cytochem.*, **17**, 303–313.

PERNOW, B. & WALDENSTRÖM, J. (1957). Determination of 5-hydroxytryptamine, 5-hydroxyindole acetic acid and histamine in thirty-three cases of carcinoid tumor (argentaffinoma). *Am. J. Med.*, **23**, 16–25.

PRINZ, R.A. & MARANGOS, P.J. (1982). Use of neuron-specific enolase as a serum marker for neuroendocrine neoplasms. *Surgery*, **92**, 887–889.

PRINZ, R.A. & MARANGOS, P.J. (1983). Serum neuron-specific enolase: a serum marker for nonfunctioning pancreatic islet cell carcinoma. *Am. J. Surg*, **145**, 77–81.

PRINZ, R.A., BERMES, Jr, E.W., KIMMEL, J.R. & MARANGOS, P.J. (1983). Serum markers for pancreatic islet cell and intestinal carcinoid tumors: a comparison of neuron-specific enolase, β-human chorionic gonadotropin and pancreatic polypeptide. *Surgery*, **94**, 1019–1023.

ROBERTS II, L.J., MARNEY, Jr, S.R. & OATES, J.A. (1979). Blockade of the flush associated with metastatic gastric carcinoid by combined histamine $H_1$ and $H_2$ receptor antagonists. Evidence for an important role of $H_2$ receptors in human vasculature. *N. Engl. J. Med.*, **300**, 236–238.

ROSANO, T.G., MEOLA, J.M. & SWIFT, T.A. (1982). Liquid-chromatographic determination of urinary 5-hydroxy-3-indoleacetic acid, with fluorescence detection. *Clin. Chem.*, **28**, 207–208.

ROSS, C., HANSEN, M.B., SCHYBERG, T. & BERG, K. (1900). Autoantibodies to crude human leucocyte interferon (IFN), native human IFN, recombinant human IFN-alpha 2b and human IFN-gamma in healthy blood donors. *Clin. Exp. Immunol.*, **82**, 57–62.

SMITH, D.B., SCARFFE, J.H., WAGSTAFF, J. & JOHNSTON, R.J. (1987). Phase II trial of rDNA alfa 2b interferon in patients with malignant carcinoid tumor. *Cancer Treat. Rep.*, **71**, 1265–1266.

SPIEGEL, R.J., SPICEHANDLER, J.R., JACOBS, S.L. & ODEN, E.M. (1986). Low incidence of serum neutralizing factors in patients receiving recombinant alfa-2b interferon (Intron A). *Am. J. Med.*, **80**, 223–228.

VEENHOF, C.H.N., DE WIT, R., TAAL, B.G., DIRIX, L.Y., WAGSTAFF, J, HENSEN, A., HULDIJ, A.C. & BAKKER, P.J.M. (1992). A dose-escalation study of recombinant interferon-alpha in patients with a metastatic carcinoid tumour. *Eur. J. Cancer*, **28**, 75–78.

VINIK, A.I., THOMPSON, N., ECKHAUSER, F. & MOATTARI, A.R. (1989). Clinical features of the carcinoid syndrome and the use of somatostatin analogue in its management. *Acta Oncol.*, **28**, 389–402.